# Protective effect of AVS073, a polyherbal formula, against UVA-induced melanogenesis through a redox mechanism involving glutathione-related antioxidant defense

**DOI:** 10.1186/1472-6882-13-159

**Published:** 2013-07-05

**Authors:** Uraiwan Panich, Thanyawan Pluemsamran, Vanida Tangsupa-a-nan, Jantanee Wattanarangsan, Rattana Phadungrakwittaya, Pravit Akarasereenont, Tawee Laohapand

**Affiliations:** 1 Department of Pharmacology, Faculty of Medicine Siriraj Hospital, Mahidol University, Bangkok 10700, Thailand; 2 Center of Applied Thai Traditional Medicine, Faculty of Medicine Siriraj Hospital, Mahidol University, Bangkok 10700, Thailand

**Keywords:** Ayurved siriraj brand wattana formula, UVA, Melanogenesis, Glutathione, Antioxidant

## Abstract

**Background:**

Ayurved Siriraj Brand Wattana formula (AVS073), a Thai herbal formula, has traditionally been used for health promotion and prevention of age-related problems. Ultraviolet A (UVA) is recognized to play a vital role in stimulation of melanin synthesis responsible for abnormal skin pigmentation possibly mediated by photooxidative stress. We thus aimed to study the inhibitory effect of AVS073 extracts on UVA-induced melanogenesis via a redox mechanism involving glutathione (GSH) synthesis and glutathione S-transferase (GST) using human melanoma (G361) cell culture.

**Methods:**

The standardization of AVS073 extracts was carried out by TLC and UHPLC to obtain fingerprinting profiles of the formula, which identified several phenolic compounds including gallic acid (GA) in the formula. Antimelanogenic actions of AVS073 (up to 60 *μ*g/ml) and GA (up to 10 *μ*g/ml) were investigated by measuring tyrosinase activity and mRNA as well as melanin level in G361 cells irradiated with UVA. Moreover, antioxidant actions of the herbal formula and GA were determined by evaluating oxidant formation and modulation of GSH-related antioxidant defenses including GSH content, GST activity and mRNA level of γ-glutamate cysteine ligase catalytic (γ-GCLC) and modifier (γ-GCLM) subunit and GST.

**Results:**

AVS073 extracts and GA, used as a reference compound, suppressed UVA-augmented tyrosinase activity and mRNA and melanin formation. In addition, pretreatment with AVS073 and GA was able to inhibit cellular oxidative stress, GSH depletion, GST inactivation and downregulation of γ-GCLC, γ-GCLM and GST mRNA in G361 cells exposed to UVA radiation.

**Conclusions:**

AVS073 formula exerted antimelanogenic effects possibly through improving the redox state by upregulation of GSH and GST. Moreover, pharmacological activity of the polyherbal formula would be attributed to combined action of different phenolic compounds present in the formula.

## Background

Herbal formula used for maintaining skin health and curing various ailments including skin problems has become increasing popular. Ayurved Siriraj Brand Wattana formula (AVS073), a Thai herbal formula, has traditionally been used for health promotion and prevention of age-related problems such as loss of appetite, gastrointestinal dysmotility and weakness. The formula is composed of 15 medicinal plants, *Piper nigrum* (L.), *Boesenbergia rotunda* (L.) Manf., *Cyperus rotundus* (L.), *Tinospora crispa*, *Terminalia chebula* Retz., *Cladogynos orientalis*, *Derris scandens* (Roxb.) Benth., *Anamirta cocculus* L., *Drypetes roxburghii* (Wall.), *Cinnamomum siamense* Craib., *Ferulaa assa-foetida* L., *Aegle maemelos* L., *Conioselinum univitatum* Trucz., *Saussurea lappa* Clark., *Cryptolepis buchanani* Roem. & Schult. Several *in vitro* and *in vivo* studies demonstrated that the extracts of AVS073’s components have a wide range of pharmacological properties, in particular, antioxidant, antiinflammatory, immunomodulating and anticancer actions [1-5]. Furthermore, the herbal components of AVS073 formula including *T. chebula*[6] and *S. Lappa*[7] were observed to yield whitening activities and inhibitory action against oxidative stress of the skin. Since having fair skin is desirable in the Eastern culture that leads to increased demand for whitening products, efforts have thus been made to develop medicinal plants as effective and safe antimelanogenic agents.

Ultraviolet (UV) radiation has been recognized as a main environmental factor contributed to hyperpigmentation or hypermelanosis through augmentation of melanin synthesis primarily controlled by tyrosinase [8]. Deleterious effects on the skin due to abnormal production of melanin have been also discussed because excess melanin could result in genotoxicity and may involve pathogenesis of malignant melanoma [9].

Previous studies have reported that UVA-induced oxidative stress through induction of oxidant formation and disruption of antioxidant defense system was correlated to elevation of melanogenesis [10,11]. Therefore, promotion of antioxidant defense capacity might be useful for the prevention of hypermelanosis. Glutathione (GSH)-related antioxidant enzymes including γ-glutamate cysteine ligase (γ-GCL), the rate-limiting enzyme in cellular GSH synthesis, and glutathione S-transferase (GST) are important detoxification enzymes vital to prevent the skin from photooxidative stress due to their abilities to maintain the redox state [12]. Since oxidative stress induced by UVA is implicated in hypermelanosis through induction of tyrosinase responsible for melanin synthesis, compounds that act as tyrosinase inhibitors and/or antioxidants could serve as a depigmenting agent [13]. Various melanin-producing cells including melanoma cell lines have been widely employed as models to investigate biology of melanogenesis and the effects of putative antimelanogenic agents [14,15]. Our study was carried out using human melanoma cell line (G361), one of lightly-pigmented cell types shown to be highly susceptible to accumulate oxidative damage mediated by UVA irradiation [16]. In order to develop AVS073 formula, a poly herbal formula, as a putative depigmenting agent, we thus aimed to investigate the antimelanogenic effect of AVS073 formula and its redox mechanisms in association with modulation of GSH-related antioxidant defense.

## Methods

### Materials

Human melanoma cell line G361 from American Type Culture Collection (ATCC, Rockville, Md, USA) was a generous gift from Assoc. Prof. Tengamnuay, Faculty of Pharmaceutical Sciences, Chulalongkorn University. Dulbecco’s modified Eagle medium (DMEM) and cell culture reagents were purchased from Invitrogen (NY, USA). The highest quality chemicals and reagents available were used and purchased from Sigma-Aldrich (MO, USA or Germany), unless otherwise indicated.

### Preparation and chromatographic fingerprint analysis of AVS073 formula extracts

**Figure 1:**
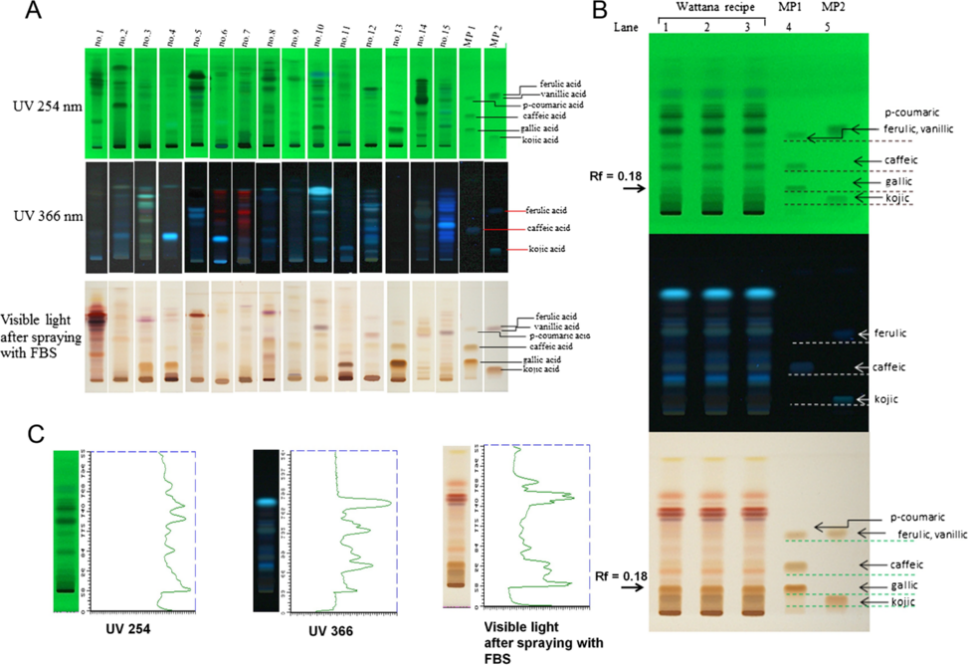
**TLC fingerprints of AVS073 and its 15 components visualized at 254 and 366 nm under UV and visible light (after spraying with fast blue salt: FBS), respectively. (A)** 15 components of AVS073 were *P. nigrum*, *B. rotunda*, *C. rotundus*, *T. crispa*, *T. chebula*, *C. orientalis*, *D. scandens*, *A. cocculus*, *D. roxburghii*, *C. siamense*, *F. assa-foetida*, *A. maemelos*, *C. univitatum*, *S. lappa*, and *C. buchanani*; lane1-15: component no. 1–15; lane16: mixed phenolic markers (MP1): gallic acid, caffeic acid and *p*-coumaric acid; lane17: mixed phenolic markers (MP2): kojic acid, vanillic acid and ferulic acid (from bottom to top). **(B)** Wattana (AVS073) formula; lane1-3; 3 replicates of AVS073; lane 4: MP1; lane 5: MP2. **(C)** Densitometric fingerprint of AVS073.

**Figure 2:**
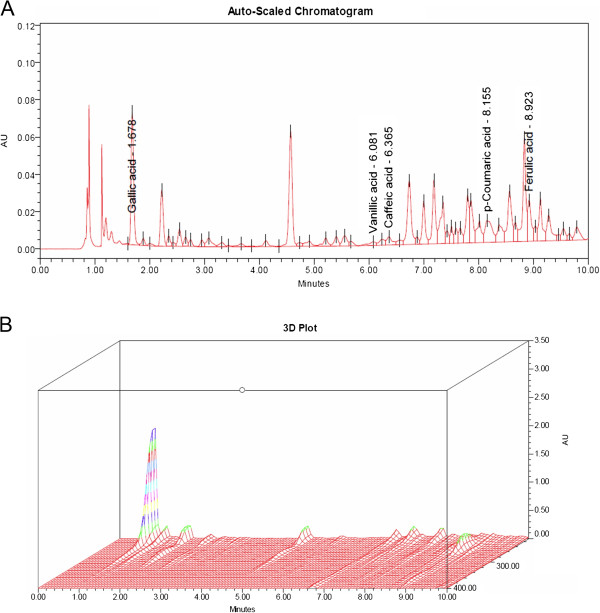
**UHPLC chromatogram of the AVS073 formula. (A)** Two-dimensional UHPLC chromatogram (UV absorbance at 280 nm). **(B)** Three-dimensional UHPLC-PDA chromatogram (range 210–400 nm).

AVS073 powder was obtained from Manufacturing Unit of Herbal Medicines and Products, manufactured under GMP by Ayurved Thamrong School, Center of Applied Thai Traditional Medicine (CATTM), Faculty of Medicine Siriraj Hospital, Mahidol University, Thailand. The whole dried AVS073 was accurately weighed (50 g) and extracted using 500 mL of 80% (v/v) ethanol as the extraction solvent. The sample solution was mixed for 10 min and centrifuged at 9,000 rpm for 10 min at 4°C. Then, the supernatant was evaporated under reduced pressure by rotary evaporator and kept frozen overnight prior to lyophilization. The lyophilized powder (5 mg) dissolved in 1 ml of methanol (50%, v/v) was used in thin layer chromatography (TLC) or ultra-high performance liquid chromatography (UHPLC) with photodiode array (PDA) detection for AVS073 analysis. The layout of the fingerprint analysis of AVS073 and its 15 components was shown in Figures 1 and 2. As shown in Figure 1, TLC-densitometric analysis was performed using a mobile phase (hexane/ethyl acetate/acetic acid = 31:14:5 v/v/v) for the separation of phenolics. TLC chromatograms showed the presence of phenolics in AVS073 extract and identification of phenolics was carried out by comparing retardation factor (Rf) values of phenolic reference markers. Gallic acid (GA) was detected in AVS073 extracts with Rf = 0.18 (Figure 1B). Moreover, two- and three- dimensional UHPLC chromatogram fingerprints were shown in Figure 2. The separations were performed on a reverse phase column (BEH C18, 1.7 μm, 2.1×100 mm, Water, Milford, MA). The mobile phases were composed of acetonitrile (B) and 0.1% o-phosphoric acid (A) using a gradient elution of 95-90% A at 0–5 min; 90-80% A at 5–8 min; 80-0% A at 8–10 min; 0-95% A at 10–11 min. The flow rate was 0.3 mL/minute. Phenolic peaks in the UHPLC fingerprint of AVS073 were analyzed and a prominent peak at a retention time (Rt) of 1.678 min was identified as GA by comparing the Rt and absorption spectrum of GA marker. Additionally, quantitative analysis of GA present in AVS073 extracts was carried out using the UHPLC method and GA content in the whole dried AVS073 extract was found to be 0.0342% w/w.

### Treatment of cells with AVS073 extracts and UVA irradiation

G361 melanoma cells were cultured in DMEM supplemented with 10% fetal bovine serum (FBS), 100 units/ml penicillin and 100 *μ*g/ml streptomycin at 37°C in a humidified atmosphere containing 5% CO_2_ (P_CO2_ = 40 Torr) (a Forma Scientific CO_2_ Water Jacketed Incubator). AVS073 extracts and GA used as a positive control were dissolved in 80% ethanol, the final concentration of which was less than 0.5% (v/v) in culture medium. G361 cells were treated with AVS073 extracts (7.5, 15, 30 and 60 *μ*g/ml) and GA (2.5, 5 and 10 *μ*g/ml) for 30 min before UV exposure for all assays and for 24 h without UV irradiation for cytotoxicity study. Cells were washed with phosphate buffered saline (PBS) and PBS was added to the cells prior to UVA (320–400 nm) exposure to avoid generation of medium-derived toxic photoproducts. For melanin content assay, the UVA dose of 16 J/cm^2^ was selected since the dose of 8 J/cm^2^ did not significantly alter melanin synthesis and both UVA doses of 8 and 16 J/cm^2^ did not affect G361 cell viability [17]. The source of UVA (330–400 nm) was xenon arc lamp (Dermalight ultrA1; Hoenle, Martinsried, Germany). After the treatment, cell lysates were prepared as previously described [17].

### Cell viability assay

In order to confirm that antimelanogenic effects of AVS073 formula was not due to cytotoxicity of the formula extracts causing a decrease in cell numbers. Cell viability was assessed using 3-(4,5-dimethylthiazol-2-yl) 2,2 diphenyltetrazolium bromide (MTT) reduction assay. Metabolically viable cells were identified by measuring the purple formazan, a product of MTT reduction. Absorbance of the purple formazan was measured at 595 nm by a spectrophotometer (SpectraMax M2 of Molecular Device, CA, USA).

### Tyrosinase activity assay

Cellular tyrosinase activity was determined by assessing the rate of L-DOPA oxidation as previously described [11]. L-DOPA was added as the substrates to each sample to initiate the enzymatic reaction at 37°C. Absorbance of dopachrome formation was measured spectrophotometrically at 475 nm every 10 min for 1 h at 37°C by a spectrophotometer. The tyrosinase activity was calculated by comparing to standard curves using tyrosinase (2,034 U/mg) and was expressed as unit/mg protein. The data are expressed as a percentage of the tyrosinase activity (unit/mg protein) of the untreated control cells without UV irradiation (100%).

### Melanin content assay

Melanin production induced by a UVA dose of 16 J/cm^2^ was assessed as previously described [11]. 1 N NaOH was used to lyse the cells and dissolve melanin, which was then determined spectrophotometrically at 475 nm. The melanin contents were calculated by comparing to a standard curve derived using synthetic melanin (0–250 *μ*g/ml) and expressed as a percentage of the melanin contents (*μ*g/mg protein) of the untreated control cells without UV irradiation (100%).

### Determination of intracellular oxidant formation

The inhibitory effects of AVS073 and GA on UVA (8 J/cm^2^)-dependent reactive oxygen species (ROS) formation in G361 cells were measured using 2′,7′-Dichlorofluorescein diacetate (DCFH-DA), a stable and non-fluorescent dye. Within the cells, DCFH-DA was hydrolyzed by esterases to non-fluorescent DCFH, which was readily oxidized by intracellular oxidants to fluorescent 2,7-dichlorofluorescein (DCF). After UVA irradiation, cells were subjected to phenol red-free DMEM containing DCFHDA (5 *μ*M) for 1 h. Then, the DCF fluorescence was monitored for 20 min at 485-nm excitation and 530-nm emission using a spectrofluorometer. The data are expressed as a percentage of intracellular oxidant formation (relative fluorescence units, RFU) of the untreated control cells without UV irradiation (100%).

### Measurement of intracellular glutathione level

A fluorometric assay based on the specific reaction of GSH with the fluorescent probe *o*-phthalaldehyde (OPA) was carried out to assess intracellular GSH as previously described [18]. After UVA treatment, cells were lysed with 6.5% (w/v) trichloroacetic acid (TCA). The assay mixture contained cell lysate, buffer (100 mM KH_2_PO_4_, 10 mM EDTA and 1 mM NaOH, pH 8.0) and 1 mg/ml OPA in methanol. The fluorescence of the GSH-OPA adduct was measured with excitation and emission wavelengths of 350 and 420 nm, respectively. The GSH levels were calculated in comparison to the standard and the results are expressed as a percentage of the GSH content (nmol/mg protein) of the untreated control cells without UV irradiation (100%).

### Measurement of glutathione-S-transferase activity

GST activity was investigated by following the kit protocol from Cayman chemical (Ann Arbor, MI). The GST catalyzed the conjugation of GSH with 1-chloro-2,4-dinitrobenzene (CDNB) to produce GSH-DNB conjugate spectrophotometrically determined at 340 nm immediately and every 30 second for 10 min. 100 mM CDNB were used to produce the reaction of either sample or positive control GST with 200 mM GSH in assay buffer. The data are expressed as a percentage of the GST activity (*μ*mol/min/mg protein) of the untreated control cells without UV irradiation (100%).

### Protein content assay

The protein content was determined using the Bio-Rad Protein Assay Kit (Bio-Rad, Germany) with bovine serum albumin (BSA) as the standard. Values for each sample are means of triplicate measurements.

### Quantitative real-time reverse transcriptase-polymerase chain reaction: determination of tyrosinase, γ-GCLC, γ-GCLM and GST mRNA expression

G361 cells pretreated with or without AVS073 (7.5, 15 and 30 *μ*g/ml) and GA (1.25, 2.5 and 5 *μ*g/ml) were exposed to UVA (8 J/cm^2^). At 2 h following UV irradiation, preparation of total RNA was carried out using the illustra RNAspin Mini RNA Isolation Kit (GE Healthcare, UK) and total RNA was reverse transcribed using the ImProm-II Reverse Transcriptase (Promega, Medison, USA) following the kit manual. 25 *μ*l of reaction mixtures contained 5 *μ*l cDNA template, 12.5 *μ*l Master Mix, 10 *μ*M forward primer (1 *μ*l), 10 *μ*M reversed primer (1 *μ*l) and 5.5 *μ*l water. Real-time PCR was carried out in triplicate for each sample on the ABI Prism 7500 Real Time PCR System (Applied Biosystems, USA). The amplification reactions were run under the following conditions: 95°C for 10 min, 40 cycles at 95°C for 15 s, 60°C for 40 s, and 72°C for 40 s. mRNA levels were determined using FastStart Universal SYBR Green Master (ROX). Primers for PCR were designed using the Primer Express software version 3.0 (Applied Biosystems, USA). Sequences of PCR primer sets for tyrosinase, γ-GCLC, γ-GCLM, GST and GAPDH (in 5′-3′ direction) were as follows:

Tyrosinase (product sizes = 114 bp)

Sense primer: TCTTCTCCTCTTGGCAGATTGTC

Antisense primer: TGTCATGGTTTCCAGGATTACG

γ-GCLC (product sizes = 160 bp)

Sense primer: GCTGTCTTGCAGGGAATGTT

Antisense primer: ACACACCTTCCTTCCCATTG

γ-GCLM (product sizes = 200 bp)

Sense primer: TTGGAGTTGCACAGCTGGATTC

Antisense primer: TGGTTTTACCTGTGCCCACTG

GST (product sizes = 72 bp)

Sense primer: CCTGTACCAGTCCAATACCATCCT

Antisense primer: TCCTGCTGGTCCTTCCCATA

GAPDH (product size = 124 bp)

Sense primer: GAAATCCCATCACCATCTTCC

Antisense primer: AAATGAGCCCCAGCCTTCTC

Amplification of a single product was verified using the melt curve analysis. The mRNA level was normalized with reference to the amount of housekeeping gene transcripts (GAPDH mRNA). The mean Ct from mRNA expression in cDNA from each sample was compared with the mean Ct from GAPDH determinations from the same cDNA samples in order to determine tyrosinase, γ-GCLC, γ-GCLM and GST mRNA. The results are expressed as fold change in gene expression calculated using the 2^−ΔΔCt^ method. For the control (untreated cells without UV irradiation), ΔΔC_t_ equals zero and 2° equals one, so that the fold change in gene expression relative to the control equals one, by definition. For the cells treated with AVS073, assessment of 2^−ΔΔCt^ determined the fold change in gene expression relative to the control.

### Statistical analysis

Values are expressed as means ± standard error of the mean (SEM) analysed from data taken from at least 3 separate experiments performed on different days. The significance of individual treatment groups in comparison to the UV-irradiated groups was determined with one-way analysis of variance (ANOVA) followed by Tukey’s *post hoc* test or independent *t*-test (Student’s; 2 populations) using Prism (GraphPad Software Inc., San Diego, CA). Values of *p* < 0.05 were considered statistically significant.

## Results

### Inhibition of UVA-induced tyrosinase activity and melanin synthesis by AVS073

**Figure 3:**
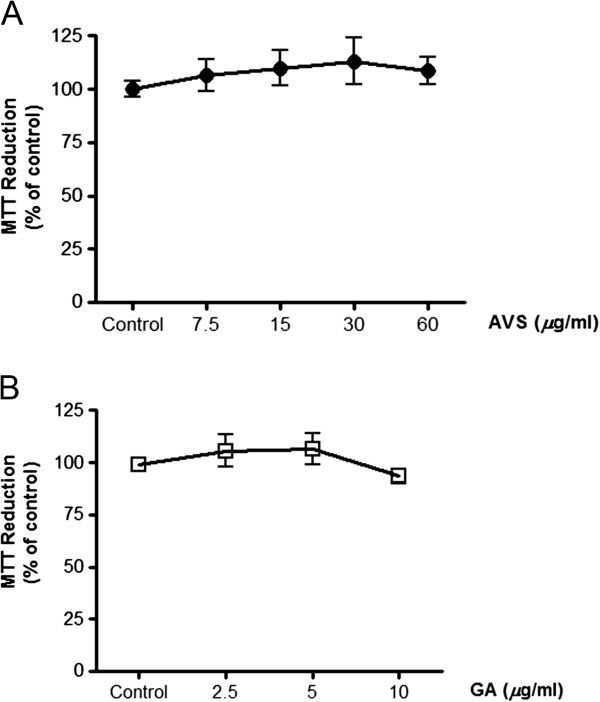
**Effects of AVS073 extracts (A) and GA (B) on cell viability without UVA irradiation.** Cell viability was expressed as a percentage of control (100%, the untreated control cells without UV irradiation).

**Figure 4:**
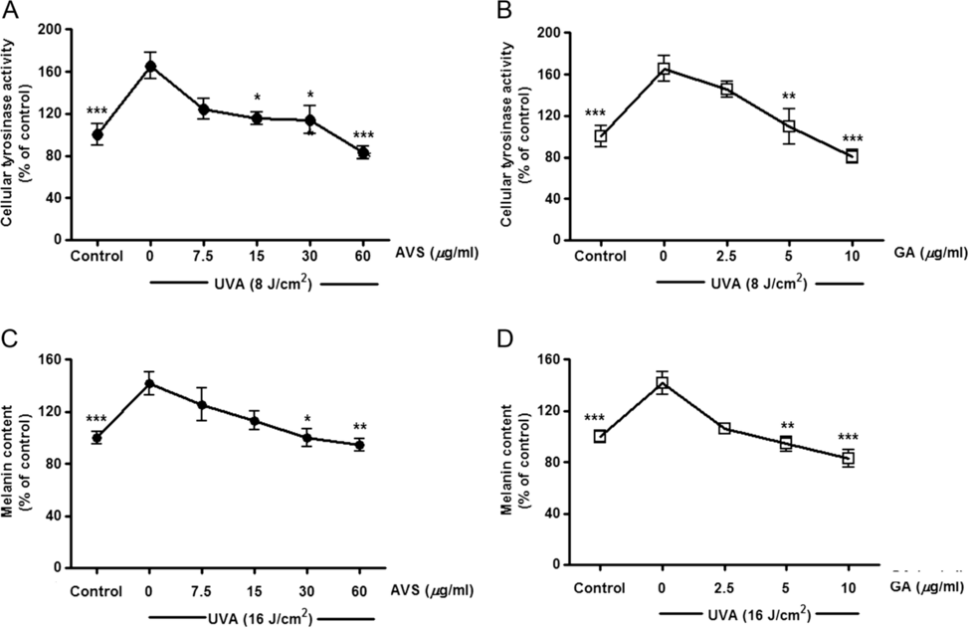
**Inhibition of UVA-induced tyrosinase activity (A and B) and melanin synthesis (C and D) by AVS073 and GA.** The tyrosinase activity and melanin production induced by a single UVA dose of 8 or 16 J/cm^2^, respectively, related to the protein concentration were expressed as a percentage of control (100%, the untreated control cells without UV irradiation). Values are expressed as mean±SEM. The statistical significance of differences between the control and UVA-irradiated cells was determined by Student’s t test and between UVA-irradiated and AVS073 extracts- or GA-treated cells by one-way ANOVA followed by Tukey’s *post hoc* test. *p < 0.05; **p < 0.01; ***p < 0.001 compared with AVS073- or GA-treated cells without UV irradiation.

**Figure 5:**
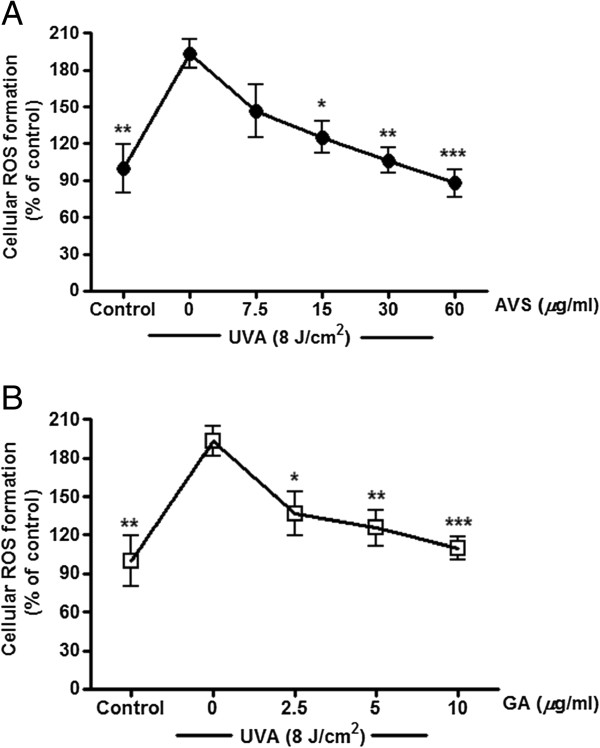
**Inhibition of UVA-induced ROS formation by AVS073 (A) and GA (B).** Oxidant formation was assessed by measurement of fluorescence of the DCF. Values are expressed as mean±SEM. The statistical significance of differences between the control and UVA-irradiated cells was determined by Student’s t test and between UVA-irradiated and AVS073 extracts- or GA-treated cells by one-way ANOVA followed by Tukey’s *post hoc* test. *p < 0.05; **p < 0.01; ***p < 0.001 compared with AVS073- or GA-treated cells without UV irradiation.

At first, cytotoxicity of the herbal formula and GA on G361 cells was assessed in order to indicate that the inhibitory effects of AVS073 and GA on melanin synthesis were not due to reduced number of cells. MTT assay demonstrated that the formula up to 60 *μ*g/ml and GA up to 10 *μ*g/ml did not affect cell viability (Figure 3A and 3B). The depigmenting effects of the herbal extracts and GA were assessed by measuring tyrosinase activity and melanin production in G361 cells irradiated with a UVA dose of 8 and 16 J/cm^2^, respectively. UVA irradiation caused 65.6 ± 11.9% (*p* < 0.001) and 42.6 ± 5% (*p* < 0.001) induction in tyrosinase activity and melanin production, respectively. However, UVA-mediated enhanced tyrosinase activity (Figure 4A and 4B) and melanin content (Figure 5C and 5D) was markedly inhibited by AVS073 extracts and GA in a concentration-dependent manner.

### Inhibition of UVA-induced ROS formation, GSH depletion and GST inactivation by AVS073

**Figure 6:**
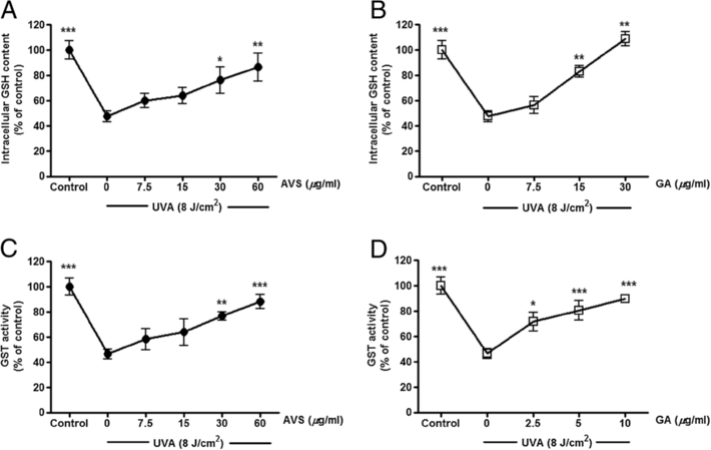
**Inhibition of UVA-induced GSH depletion (A and B) and GST inactivation (C and D) by AVS073 and GA.** GSH level and GST activity were determined by measurement of fluorescence of the GSH-OPA adduct and absorbance of the GSH-CDNB, respectively. The GSH content and GST activity related to the protein concentrations were expressed as a percentage of control (100%, untreated cells without UV irradiation). Values are expressed as mean±SEM. The statistical significance of differences between the control and UVA-irradiated cells was determined by Student’s t test and between UVA-irradiated and AVS073 extracts- or GA-treated cells by one-way ANOVA followed by Tukey’s *post hoc* test. *p < 0.05; **p < 0.01; ***p < 0.001 compared with AVS073- or GA-treated cells without UV irradiation.

The redox mechanisms associated with antimelanogenic effects of AVS073 were assessed by investigating GSH-related antioxidant defenses including GSH level and GST activity. Irradiation of G361 cells with UVA (8 J/cm^2^) caused a substantial elevation in intracellular oxidant production by 93.6 ± 11.7% (*p* < 0.01) in irradiated cells compared to nonirradiated cells. In contrast, pretreatment of cells with AVS073 (15–60 *μ*g/ml) (Figure 5A) and GA (2.5-10 *μ*g/ml) (Figure 5B) resulted in a dose-dependent inhibition of UVA-induced oxidant generation. Moreover, a significant decline in GSH content by 52.5 ± 7.2% (*p* < 0.001) and GST activity by 53.5 ± 9.4% (*p* < 0.001) (Figure 6) was observed in irradiated G361 cells compared to nonirradiated cells. Nevertheless, AVS073 extracts and GA were observed to protect against the loss of GSH (Figure 6A and 6B) and inactivation of GST (Figure 6C and 6D) in a concentration-dependent manner.

### Inhibition of UVA-induced upregulation of tyrosinase mRNA and downregulation of γ-GCLC, γ-GCLM and GST by AVS073

**Figure 7:**
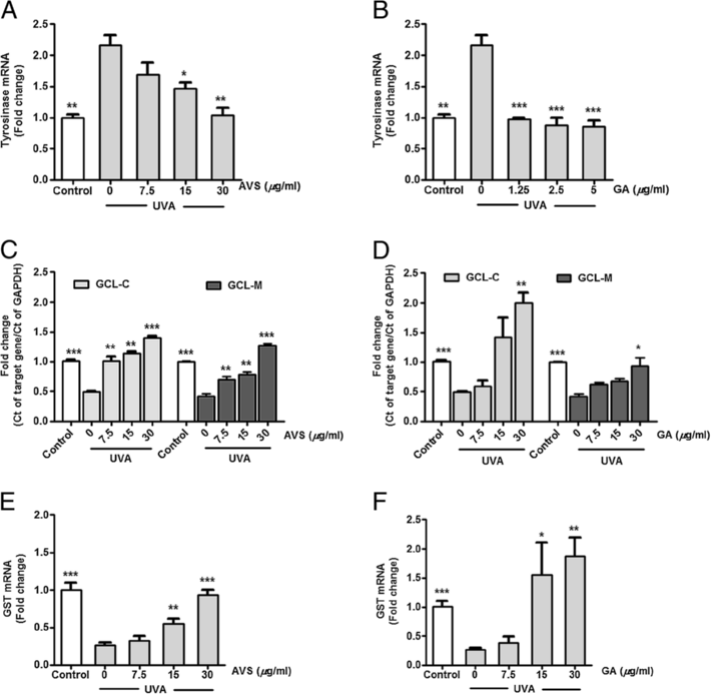
**Inhibition of UVA-induced upregulation of tyrosinase mRNA (A and B) and downregulation of γ-GCLC and γ-GCLM (C and D) as well as GST (E and F) by AVS073 and GA.** The data shown as the fold change in gene expression normalized to GAPDH and relative to the control sample. Values given are mean±SEM. The statistical significance of differences between the control and UVA-irradiated cells was determined by Student’s t test and between UVA-irradiated and AVS073 extracts- or GA-treated cells by one-way ANOVA followed by Tukey’s *post hoc* test. *p < 0.05; **p < 0.01; ***p < 0.001 compared with AVS073- or GA-treated cells without UV irradiation.

The quantitative analysis of gene expression changes was carried out in order to investigate the effects of AVS073 (7.5-30 *μ*g/ml) and GA (1.25-5 *μ*g/ml) on increased melanogenesis at mRNA level at 2 h after irradiation. In agreement with the data observed in the study of tyrosinase activity, a rise in tyrosinase mRNA expression (2.2 ± 0.2-fold change; *p* < 0.01) was observed in response to UVA irradiation (8 J/cm^2^), although AVS073 (Figure 7A) and GA (Figure 7B) significantly diminished tyrsoinase mRNA levels. Furthermore, while UVA exposure led to reduction of mRNA levels of γ-GCLC (0.51 ± 0.01-fold decrease; *p* < 0.001), γ-GCLM (0.59 ± 0.04-fold decrease; *p* < 0.001) and GST (0.74 ± 0.04-fold decrease; *p* < 0.001), upregulation of their mRNA was detected in the cells pretreated with AVS073 extracts (Figure 7C and 7E) and GA (Figure 7D and 7F).

## Discussion and conclusion

Potential medicinal plants containing antioxidant properties have gain remarkable attention in development of novel whitening products since UVA-dependent oxidative stress is recognized to aggravate hyperpigmentation, a common skin problem that physically and emotionally affects mostly Asian women. Assays for tyrosinase activity and melanin production are common screening methods used to determine antimelanogenic effects of putative compounds. Additionally, medicinal plants possessing antioxidant properties play a crucial role in the protection of photooxidative stress through different redox mechanisms including quenching free radicals and improving antioxidant defense capacity. Our observation suggested that AVS073 formula at non-cytotoxic concentrations was able to substantially inhibit UVA-dependent increased melanin production and tyrosinase activity and mRNA expression in G361 cells. The antioxidant roles of AVS073 in regulation of melanogenesis were also investigated in our study. We previously demonstrated that ROS production in response to UV irradiation correlated with upregulation of tyrosinase and melanin synthesis in human melanoma cells [11]. In this study, the extracts of AVS073 formula were found to suppress UVA-induced ROS formation in relation to modulation of GSH-related antioxidant defense. GSH-related antioxidant defense including GSH-GCL system and GST is important for the cells to cope with oxidative stress as their actions involve restoration of intracellular redox homeostasis and detoxification of UV-dependent generation of oxidants implicated in skin damage [19]. In addition, it is well recognized that GSH is capable of modulating melanogenesis and the opposite regulation of tyrosinase by GSH has been observed in previous studies suggesting that promotion of GSH content was associated with a decrease in melanogenesis in human melanoma cells [11]. Since insufficient elimination of oxidants appears to induce tyrosinase activity and melanin production, GSH-related detoxifying antioxidants may thus play a role in the regulation of melanogenesis and/or neutralization of toxic intermediates generated during melanogenic process [20]. Pretreatment of irradiated G361 cells with the AVS073 extracts was found to upregulate GSH levels and GST activity in our study. We then determined whether AVS073 also affected the transcriptional regulation of GSH biosynthesis and GST and found that the extracts were able to protect against UVA-induced downregulation of γ-GCLC, γ-GCLM and GST mRNA. Nevertheless, further investigations using physiologically relevant skin models, e.g., primary human melanocytes, are required since various pigment cell types have different redox states that might affect melanin biosynthesis in response to UVA exposure.

In addition, the fingerprint profile of the AVS073 extracts was carried out using TLC and UHPLC analysis to ensure consistent quality of preparations of the herbal extracts studied. While the chromatogram showed the presence of several phenolic compounds including GA in the AVS073 formula, which was then used as the positive control to screen antimelanogenic effects of the herbal formula, GA content in the AVS073 extracts was observed to be 0.0342% w/w, which was very low, and its concentration at 5 *μ*g/ml was required to achieve a significant inhibition of UVA-induced tyrosinase activity (Figure 4B) and melanin content (Figure 4B) in G361 cells. Therefore, GA could not be solely a phenolic compound contributed to biological activity of the AVS073 extracts and antimelanogenic effect of the formula may be attributed to combined effect of multiple phytochemicals present in the formula. It is thus important to further identify active components and/or ingredients of the whole formula and its constituent herbs responsible for the pharmacological activities of the AVS073 formula. There is considerable interest in screening of botanical products including herbal formulas possessing antioxidant properties accountable for depigmenting activity in order to develop effective and safe whitening agents. Previous *in vitro* and *in vivo* studies have shown that *P. nigrum*[21], *C. rotundus*[1], *T. chebula*[22], *D. scandens*[23], *F. asafoetida*[24], *S. lappa*[3,7], *C. buchanani*[23] provided powerful free radical scavenging activities and/or restored antioxidant defense system including GSH or GST at the cellular and molecular level. *T. chebula* fruit also exhibited inhibitory effects on UVB-induced oxidative stress and damage of human epidermal keratinocytes [22]. Furthermore, in agreement with our observation on the antimelanogenic effects of GA on G361 and B16F10 melanoma cells [16], GA, an important phenolic acid present in AVS073 formula (Figure 2A), was also shown to protect against UVA-induced melanogenesis in this study. It may be possible that the antimelanogenic effects of AVS073 formula may be attributed to antioxidant actions of several phenolic compounds including GA in neutralizing ROS generated by UV but not direct effects since treatment of G361 cells with the herbal extracts alone at the highest concentration tested (60 *μ*g/ml) for 30 min without UVA irradiation did not significantly affect tyrosinase activity, melanin content, ROS formation, GSH level and GST activity (Table 1).

**Table 1:** **The effects of AVS073 extracts alone at the highest concentration tested (60** ***μ*g/ml) on tyrosinase activity, melanin content, ROS formation, GSH level and GST activity in G361 cells compared to control group**

**Assay**	**% of control**		***p*-value**
	**Unirradiated and untreated control**	**AVS073 alone without irradiation**	
*Tyrosinase activity*	100 ± 10.3	99.5 ± 5.5	> 0.05
*Melanin content*	100 ± 4.7	110.06 ± 15.2	> 0.05
*ROS formation*	100 ± 7.8	87.1 ± 9.3	> 0.05
*GSH level*	100 ± 7.1	94.36 ± 5.5	> 0.05
*GST activity*	100 ± 6.7	106.24 ± 6.6	> 0.05

Results are expressed as mean ± SEM.

In summary, the AVS073 formula, a Thai herbal formula, provided inhibitory effects on melanogenesis in association with the redox mechanism involving upregulation of GSH biosynthesis as well as GST activity and mRNA. Our observation could give pharmacological evidence for the traditional use of the herbal formula and further studies are warranted to develop this formula and/or to identify active ingredients as effective depigmenting agents.

## Abbreviations

AVS073: Ayurved siriraj brand wattana formula™; BSA: Bovine serum albumin; CDNB: 1-Chloro-2,4-dinitrobenzene; DCFH-DA: 2′, 7′-Dichlorofluorescein diacetate; DMEM: Dulbecco’s modified eagle medium; FBS: Fetal bovine serum; GA: Gallic acid; γ-GCLC: γ-Glutamate cysteine ligase catalytic subunit; γ-GCLM: γ-Glutamate cysteine ligase modifier subunit; GMP: Good manufacturing practice; GSH: Glutathione; GST: Glutathione S-transferase; MP: Mixed phenolic markers; MTT: 3-(4,5-Dimethylthiazol-2-yl)-2,5-diphenyltetrazolium bromide; OPA: *o*-Phthalaldehyde; PBS: Phosphate buffered saline; Rf: Retardation factor; ROS: Reactive oxygen species; Rt: Retention time; TCA: Trichloroacetic acid; TLC: Thin layer chromatography; UHPLC: Ultra-high performance liquid chromatography; UV: Ultraviolet.

## Competing interests

The authors declare that they have no completing interests.

## Authors’ contributions

UP designed the study, analyzed the data and wrote the manuscript. TP and VT performed the experimental studies of cellular melanogenesis and antioxidant defense. JW and RP carried out the chromatographic fingerprint analysis. PA wrote the manuscript. TL conceived the study and provided the herbal samples. All authors read and approved the final manuscript.

## Pre-publication history

The pre-publication history for this paper can be accessed here:

http://www.biomedcentral.com/1472-6882/13/159/prepub
